# Electron diffraction and dark-field TEM for structural analysis of 2D van der Waals materials

**DOI:** 10.1186/s42649-025-00117-3

**Published:** 2025-11-11

**Authors:** Byunghyun Kim, Daesung Park, Siwon Jeong, Hyobin Yoo

**Affiliations:** 1 Department of Materials Science and Engineering, Seoul National University, Seoul, 08826 Republic of Korea; 2 Department of Physics, Sogang University, Seoul, 04107 Republic of Korea; 3 Research Institute of Advanced Materials, Seoul National University, Seoul, 08826 Republic of Korea

**Keywords:** 2D van der Waals materials, Electron diffraction, Transmission electron microscopy, Dark-field imaging

## Abstract

Two-dimensional (2D) van der Waals materials possess structural degrees of freedom that set them apart from conventional bulk crystals and strongly influence their physical properties. Such freedom, enabled by the weak interlayer bonding, permits stacking, twisting, and lateral sliding of layers, leading to structural variations such as out-of-plane corrugations, layer-number–dependent electronic and optical responses, and interlayer registry variations that produce stacking domains with distinct functionalities. Capturing and understanding these variations is essential for linking structure to function. Transmission electron microscopy (TEM) offers complementary approaches for this purpose: electron diffraction provides quantitative crystallographic fingerprints, while dark-field (DF) imaging translates selected diffraction information into spatial maps of local structure. When combined, these techniques can resolve complex structural modulations across multiple length scales and under diverse experimental conditions. Recent advances have extended diffraction and DF imaging into in-situ and operando regimes, enabling real-time observation of domain reconfiguration, phase transitions, and polarization switching under external stimuli. This review discusses how these methods are applied to 2D van der Waals materials to reveal structural degrees of freedom and illustrates their unique capability to directly connect structural evolution to functional behavior.

## Introduction

Two-dimensional (2D) van der Waals materials have transformed the landscape of materials science by offering atomic-scale control over structure and properties. The weak interlayer bonding characteristic of these materials allows individual atomic planes to be isolated and manipulated in ways that are not possible in conventional bulk crystals (Novoselov et al. 2004; Zhang et al. 2005). This structural flexibility facilitates stacking (Dean et al. 2010; Britnell et al. 2012a, b; Haigh et al. 2012; Geim and Grigorieva 2013; Gong et al. 2014; Hong et al. 2014; Tongay et al. 2014; Cui et al. 2015), twisting (Li et al. 2010; Yankowitz et al. 2012; Dean et al. 2013; Liu et al. 2014a; Woods et al. 2014; Kim et al. 2016), and lateral sliding of layers (Li and Wu 2017; Yasuda et al. 2021; Vizner Stern et al. 2021; Wang et al. 2022a; Weston et al. 2022; Meng et al. 2022; Ko et al. 2023; Sui et al. 2023), enabling access to a rich configurational phase space. These degrees of freedom not only create opportunities for material design but also fundamentally alter electronic (Bistritzer and MacDonald 2011; Luican et al. 2011; Hunt et al. 2013; Ponomarenko et al. 2013; Liu et al. 2020; Regan et al. 2020; Wang et al. 2020), optical (Britnell et al. 2013; Fang et al. 2014; Ceballos et al. 2015; Rivera et al. 2015, 2016; Sunku et al. 2018; Jin et al. 2019), mechanical (Lee et al. 2008; Hod 2012; Liu et al. 2014b; Jiang and Park 2014), and ferroic properties (Li and Wu 2017; Yasuda et al. 2021; Vizner Stern et al. 2021; Wang et al. 2022a; Weston et al. 2022; Meng et al. 2022; Sui et al. 2023; Zhong et al. 2017; Gong et al. 2019; Sharpe et al. 2019; Zheng et al. 2020; Wu et al. 2022). With the discovery of correlated and topological phases in twisted bilayer graphene (Cao et al. 2018a, b; Yankowitz et al. 2019; Saito et al. 2020; Park et al. 2021) and ferroelectricity in twisted hexagonal boron nitride (h-BN) (Yasuda et al. 2021; Vizner Stern et al. 2021) and transition metal dichalcogenides (TMDs) (Wang et al. 2022a; Weston et al. 2022; Meng et al. 2022; Ko et al. 2023), structural degrees of freedom have become as critical as chemical composition in determining functionality.

Understanding and exploiting this structural tunability requires precise, spatially resolved structural analysis tools. Transmission electron microscopy (TEM) offers two complementary modes: electron diffraction and dark-field (DF) imaging. Electron diffraction provides quantitative crystallographic information, revealing lattice symmetries (Song et al. 2010; Geng et al. 2012; Gao et al. 2015a; Zhou et al. 2015; Ahn et al. 2018), three-dimensional distortions (Meyer et al. 2007a, b; Kirilenko et al. 2011; Thomsen et al. 2017), strain states (Ahn et al. 2017; Kim et al. 2019; Thodkar et al. 2024), and interlayer orientations with high angular precision (Thodkar et al. 2024; Brown et al. 2012; Yuk et al. 2014; Dumcenco et al. 2015; Latychevskaia et al. 2019; Sung et al. 2019, 2022a; Yoo et al. 2019). DF imaging uses selected diffraction spots to transform this reciprocal-space information into real-space contrast, enabling the direct visualization of orientation domains (Huang et al. 2011; Kim et al. 2011; Rasool et al. 2011; Tsen et al. 2012; Lee et al. 2013), stacking boundaries (Ko et al. 2023; Yoo et al. 2019; Alden et al. 2013; Lin et al. 2013; Butz et al. 2014; Park et al. 2025), and stacking sequences (Brown et al. 2012; Yoo et al. 2019; Alden et al. 2013; Lin et al. 2013; Butz et al. 2014; Ping and Fuhrer 2012). By combining these approaches, one can directly connect diffraction-based structural fingerprints with spatial maps of local variations, capturing subtle structural modulations that are otherwise difficult to detect. This integration makes TEM uniquely capable of resolving the complex structural landscape of 2D materials across multiple length scales and under diverse experimental conditions.

Moreover, the combined use of electron diffraction and DF imaging is highly adaptable to in-situ and operando experiments. Diffraction patterns acquired during thermal cycling, electrical biasing, or mechanical deformation can track changes in lattice constants, strain distributions, phases, and stacking configurations in real time (Gao et al. 2015b; Fei et al. 2016; Zheng et al. 2022). At the same time, DF imaging can map the spatial evolution of these transformations, revealing how domains nucleate, migrate, or reorganize under external stimuli (Ko et al. 2023; Alden et al. 2013; Lee et al. 2023; Winkle et al. 2024; Zhang et al. 2024). This dual capability is particularly valuable as it offers a direct link between structural changes and functional responses with both spatial and temporal resolution.

In this review, we discuss the application of the combined electron diffraction and DF TEM imaging to the structural analysis of 2D van der Waals materials. We emphasize how these techniques reveal crystallinity, three-dimensional structural modulation, stacking order variations from bilayer to multilayer systems, and structural symmetry-related phenomena. We also examine their use in in-situ and operando studies, where structural dynamics under external stimuli can be captured in real time. By covering both the fundamental principles and recent advances, this review aims to provide a comprehensive framework for utilizing electron diffraction and DF imaging as complementary tools for understanding and engineering the structural degrees of freedom in 2D materials.

## Large-area orientation and grain boundary analysis

Grain boundaries and rotational grains play a critical role in determining the macroscopic behavior of 2D materials, especially those grown by scalable techniques including chemical vapor deposition (CVD) method. In such samples, rotational disorder, small-angle and high-angle grain boundaries introduce variations in carrier mobility (Tsen et al. 2012; Yazyev and Louie 2010; Yu et al. 2011; Song et al. 2012; Clark et al. 2013; van der Zande et al. 2013; Ly et al. 2016), strain accumulation (Azizi et al. 2014; Elibol et al. 2017; Han et al. 2018a, b; Xie et al. 2018), and interfacial reactivity (Kim et al. 2014; Yasaei et al. 2014; Rong et al. 2015; Fan et al. 2018; Zhu et al. 2019). Capturing these features over statistically relevant areas while maintaining crystallographic sensitivity requires a technique that bridges the gap between atomic-resolution imaging and bulk-scale structural probes.

**Fig. 1:**
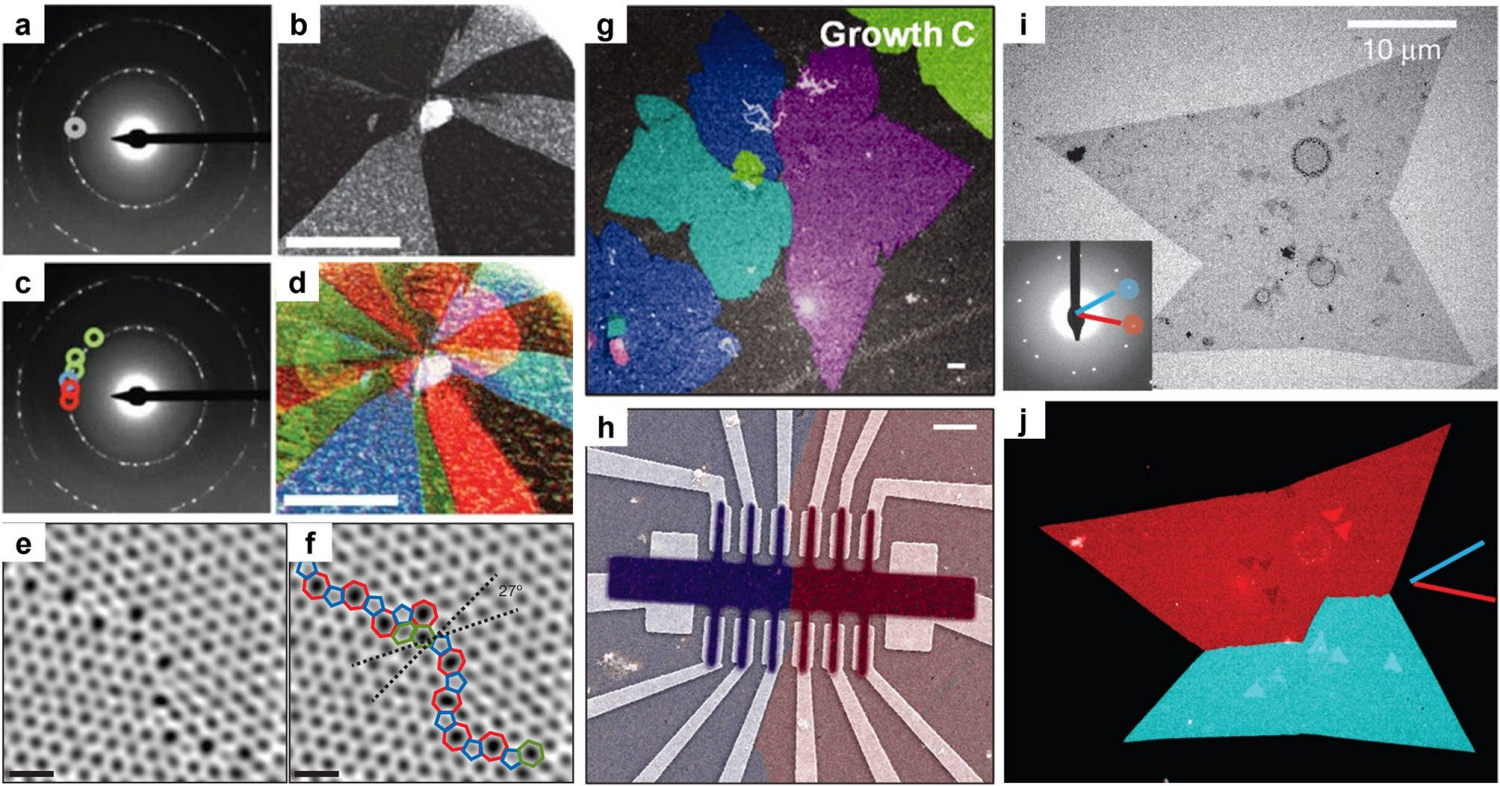
Grain orientation and grain boundary analysis. (**a-b**) In graphene with differently oriented grains, the SAED pattern (**a**) and the DF image (**b**) (Scale bar, 500 nm) obtained from the circled spot in (**a**) are shown. (**c-d**) Multiple diffraction spots selected in SAED (**c**) were used to construct the corresponding color-coded image of graphene (**d**) (Scale bar, 500 nm). **(e-f)** ADF-STEM image of two grains and their boundary (**e**), with the pentagon–heptagon pairs highlighted in (**f**). Scale bar, 5 Å. Adapted with permission (Huang et al., 2011). Copyright Springer Nature. (**g-h**) Color-coded DF image (**g**) of polycrystalline graphene consisting of grains ~ 50 μm in size, and the device fabricated on a single grain boundary (**h**). Scale bars, 1 μm. Adapted with permission (Tsen et al., 2012). Copyright the American Association for the Advancement of Science. (**i-j**) Bright-field (BF) image (**i**) of a monolayer MoS₂ consisting of two grains, along with its SAED pattern (inset), and the color-coded DF image (**j**) reconstructed from selected diffraction spots in SAED. Adapted with permission (van der Zande et al., 2013). Copyright Springer Nature

DF TEM meets this need by enabling large-area orientation mapping through Bragg spot–filtered imaging. In a typical workflow, a selected area electron diffraction (SAED) pattern is acquired from the region of interest (Fig. 1a), and an objective aperture is used to isolate a specific diffraction vector. The resulting DF image (Fig. 1b) displays the spatial distribution of lattice planes corresponding to that diffraction condition. By repeating this process across multiple non-equivalent Bragg peaks, a complete orientation map can be constructed. Color-coding the DF images obtained from different spots enables visualization of grain orientation, misorientation angles, and boundary distributions with sub-micrometer spatial resolution (Fig. 1c, d) (Huang et al. 2011; Kim et al. 2011; Tsen et al. 2012; Lee et al. 2013).

This approach has been particularly powerful in monolayer and few-layer graphene, where CVD growth often results in a patchwork of rotational grains (Rasool et al. 2011; Lee et al. 2013). DF TEM has revealed large-angle grain boundaries separating grains misoriented by 20° to 30°, as well as low-angle boundaries formed by wrinkle-induced strain during transfer. Mapping these features over tens of microns enables direct correlation with electrical measurements such as local conductivity and carrier scattering. In one example, DF TEM (Fig. 1c, d) was combined with atomic-resolution annular dark-field scanning TEM (ADF-STEM) (Fig. 1e, f) to identify 27° grain boundaries composed of periodic pentagon–heptagon dislocation cores in monolayer graphene (Huang et al. 2011). In related work, Tsen et al. (2012) used DF TEM (Fig. 1g) to locate and resolve grain boundaries in polycrystalline graphene before fabricating Hall bar devices intersecting these boundaries with sub-50 nm alignment accuracy (Fig. 1h). Electrical transport across boundaries with known structures showed that well-connected, atomically sharp boundaries had only slightly reduced conductance compared to single grains, whereas poorly stitched boundaries containing amorphous or overlapping regions could be up to an order of magnitude more resistive.

Beyond graphene, DF TEM orientation mapping has been applied to other 2D semiconductors such as CVD-grown monolayer MoS_2 _(van der Zande et al. 2013). In that work, large triangular single-crystal grains exceeding 100 μm were confirmed to be continuous, while polycrystalline films were resolved into distinct grains separated by tilt or mirror twin boundaries. DF TEM (Fig. 1i, j), combined with atomic-resolution STEM, showed that tilt boundaries were stitched by lines of non-hexagonal rings and that mirror twin boundaries produced distinct contrast due to the sublattice asymmetry of Mo and S atoms. Correlation with optical and transport measurements revealed that mirror twins caused photoluminescence quenching but little change in in-plane conductivity, while certain tilt boundaries enhanced photoluminescence but slightly reduced electrical conductance. These results highlight how DF TEM, when combined with complementary high-resolution imaging, can directly connect grain boundary structures to the optical and electronic responses of 2D semiconductors.

In addition to DF TEM orientation mapping, the accompanying SAED provides further insight into the crystallographic structure. While the angular distribution of diffraction spots in SAED reveals the in-plane orientation of individual grains, the intensity ordering of these spots provides information about the out-of-plane lattice orientation or equivalently the 2D chirality. In low-symmetry monolayers such as triclinic ReS_2_, this chirality originates from the loss of inversion symmetry when a monolayer is supported on a substrate, resulting in two enantiomeric configurations that differ in whether the layer faces upward or downward with respect to the substrate. Therefore, the diffraction spots of the $\:\left\{100\right\}$ planes also exhibit chirality, with their intensity sequence appearing in either a clockwise or a counterclockwise order. Their handedness can thus be directly identified from SAED patterns (Chen et al. 2024; Jiang et al. 2025). These studies demonstrate that electron diffraction provides diverse crystallographic information across a wide range of 2D materials.

## Quantification of three-dimensional structural modulation

**Fig. 2:**
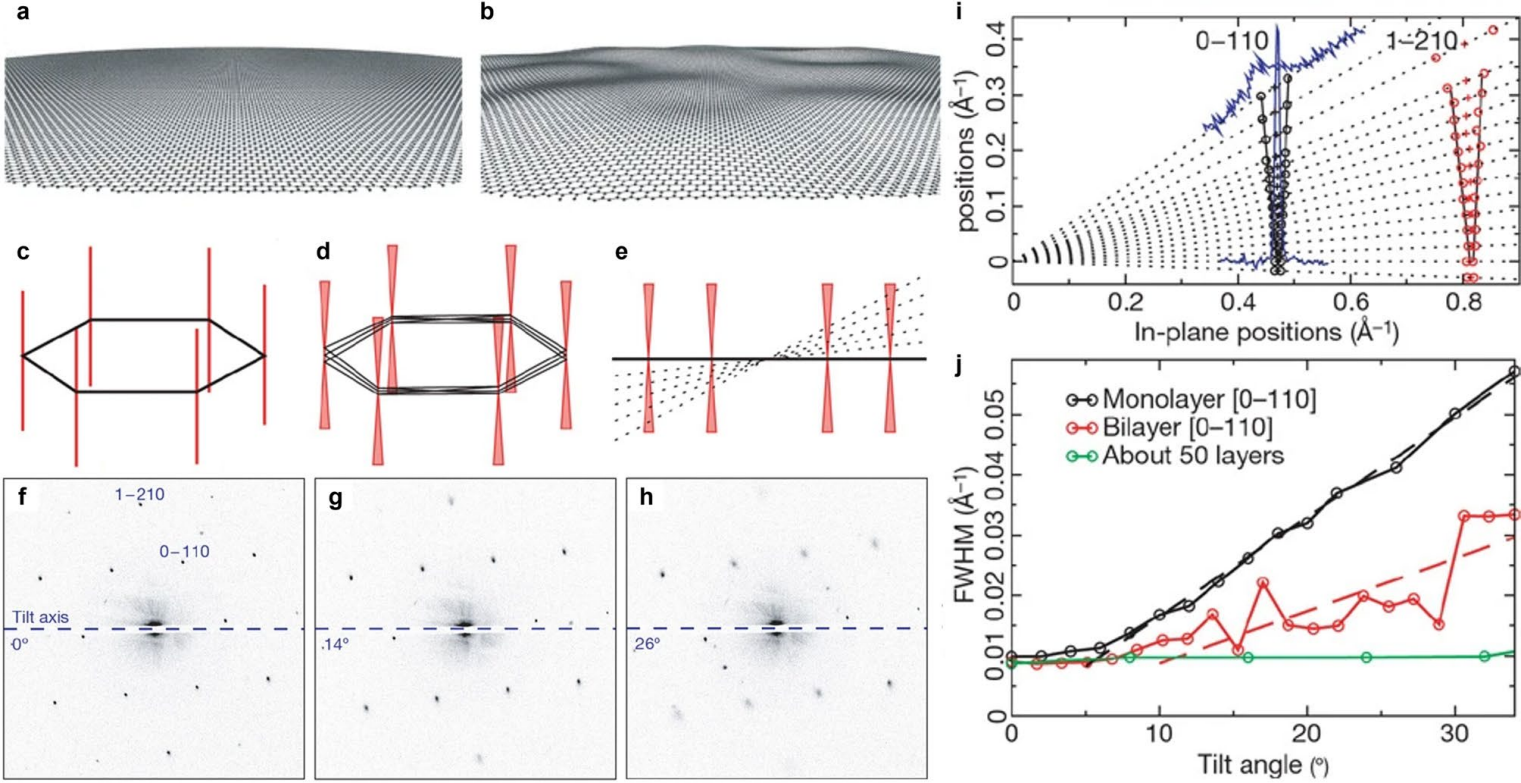
Diffraction analysis of corrugated graphene. (**a–b**) Schematic illustrations of flat graphene (**a**) and corrugated graphene (**b**). (**c–d**) Reciprocal rods corresponding to flat graphene (**c**) and corrugated graphene (**d**). (**e**) Intersection between the Ewald sphere (dashed line) and reciprocal rods under specimen tilt. (**f-g**) Evolution of diffraction spots with graphene tilt angles of 0° (**f**), 14° (**g**), and 26° (**h**). (**i**) Intensity profiles of diffraction peaks as a function of graphene tilt. (**j**) Broadening of diffraction peaks with increasing tilt angle. Adapted with permission (Meyer et al. 2007b). Copyright Springer Nature

Unlike conventional bulk crystals, 2D materials can exhibit significant out-of-plane distortions caused by thermal fluctuations, intrinsic rippling, or strain relaxation (Meyer et al. 2007b; Fasolino and Los 2007; Ishigami et al. 2007; Obraztsov et al. 2007; Xu et al. 2014; Bronsgeest et al. 2015) (Fig. 2a, b). These corrugations are not merely morphological features but are closely linked to the fundamental stability and functionality of the material. For example, surface undulations can influence electron-phonon coupling (Mariani and Oppen 2008; Castro et al. 2010; Laitinen et al. 2014; Ng et al. 2022), modulate the band structure (Mohiuddin et al. 2009; Pereira and Castro Neto 2009; Conley et al. 2013; He et al. 2016), and thereby affect device performance. Quantifying such out-of-plane deformations with high precision is therefore a central challenge in the field.

Electron diffraction provides a sensitive and non-destructive means to probe corrugations through analysis of the shape and broadening of Bragg reflections (Meyer et al. 2007a, b; Kirilenko et al. 2011; Thomsen et al. 2017). In TEM, the diffraction pattern corresponds to the intersection of the reciprocal lattice with the nearly flat Ewald sphere. For a perfectly flat 2D crystal, the reciprocal lattice consists of elongated rods perpendicular to the plane, which intersect the Ewald sphere as sharp Bragg spots regardless of specimen tilt (Fig. 2c). In contrast, a corrugated membrane has a distribution of local surface normals, which modifies the reciprocal lattice from straight rods to cone-like shapes (Fig. 2d). As the specimen is tilted, the intersection of the Ewald sphere with these cones produces Bragg spots that broaden (Fig. 2e and f-h). By quantifying this tilt-dependent broadening (Fig. 2i), one can extract the statistical distribution of local tilt angles (Fig. 2j).

Tilt-series diffraction experiments on suspended monolayer graphene have shown cone-like broadening with cone angles of approximately 8° to 11°, corresponding to surface-normal variations of about 5° to 6°. In bilayer graphene, the variation in surface normal was found to be 2° presumably due to the increase in the membrane stiffness (Meyer et al. 2007a, b).

While analogous tilt-series diffraction broadening analyses are well established for graphene, extending the same protocol to other 2D materials such as h-BN (Pan et al., 2012), TMDs (Brivio et al. 2011; Shao et al. 2022), and In_2_Se_3 _(Zheng et al., 2022) remains an opportunity. Given their differing elastic constants and common processing routes, including substrate-supported growth and polymer-assisted transfer, one would expect material-specific corrugation signatures and wrinkle-induced distortions. Systematic, diffraction-based comparisons across layer numbers, substrates, and processing conditions would help quantify how process history shapes the final morphology.

## Layer number identification in 2D materials

The number of atomic layers in 2D materials critically influences their electronic (Zhang et al. 2009; Li et al. 2014a; Xi et al. 2016; Zhou et al. 2016), optical (Ferrari et al. 2006; Nair et al. 2008; Li et al. 2013; Zhang et al. 2014), and ferroic properties (Meng et al. 2022; Gong et al. 2017; Huang et al. 2017; Deng et al. 2018). For example, TMDs including MoS_2_, MoSe_2_, WS_2_, and WSe_2_ exhibit a thickness dependent transition from a direct to an indirect bandgap (Mak et al. 2010; Splendiani et al. 2010; Jin et al. 2013; Zhao et al. 2013; Li et al. 2014b), while multilayer structures show interlayer-registry–driven phenomena such as emergent phases and symmetry-dependent functionalities (Yasuda et al. 2021; Vizner et al. 2021; Wang et al. 2022a; Weston et al. 2022; Tsen et al. 2015; Yoshida et al. 2015; Fei et al. 2018) . In TEM, the layer number can be determined without atomic-resolution imaging by exploiting the sensitivity of specific diffraction intensities to the number of scattering planes. This relationship is governed by the crystal’s structure factor, which reflects the arrangement of atoms within the unit cell and incorporates the interference between electron waves scattered from successive layers.

**Fig. 3:**
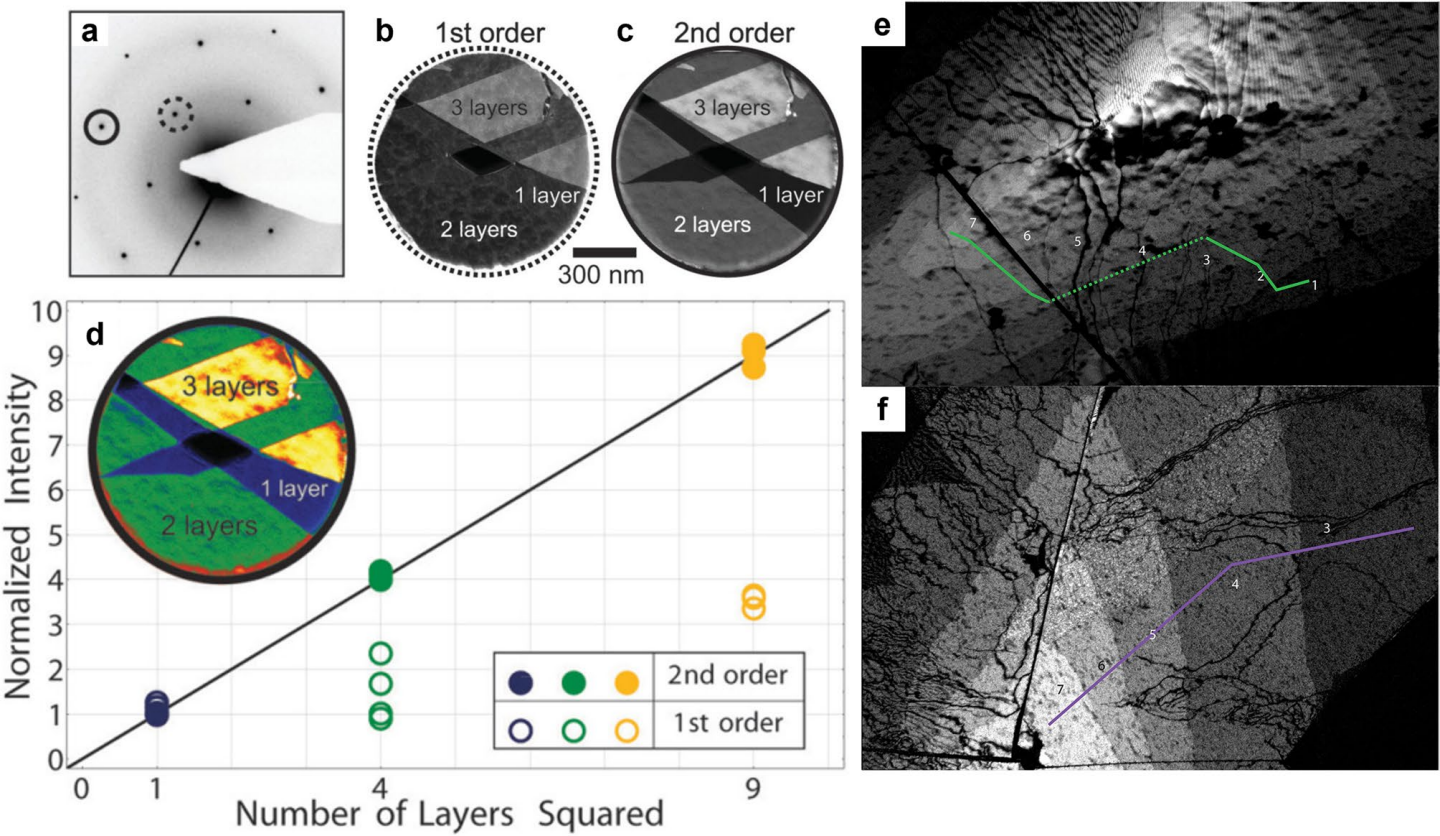
Layer number identification of graphene by DF TEM. (**a-c**) SAED of few-layer graphene (**a**), along with the first-order DF image from the dashed circle (**b**) and the second-order DF image from the solid circle (**c**). (**d**) Intensity variation in the first- and second-order DF images as a function of the number of layers; inset shows color-coded DF images of the same region as in (**b**) and (**c**). Adapted with permission (Shevitski et al., 2013). Copyright American Physical Society. **(e-f**) Second-order DF images of few-layer graphene, up to 7 layers. Adapted with permission from (Ping et al., 2012). Copyright American Chemical Society

For certain layered crystals with specific symmetries, such as the hexagonal lattice of graphene, particular orders of Bragg reflections can be selected to determine the number of layers with high accuracy. In graphene, for example, the second-order Bragg peaks ($\:g=11\bar{2}0$) exhibit an intensity that scales approximately with the square of the number of layers (∝N^2^) under kinematic conditions. This strong thickness dependence enables robust layer-number identification in DF TEM images. Selecting this reflection with the objective aperture in DF TEM mode produces contrast in which brighter regions correspond to thicker layers, allowing monolayer, bilayer, and few-layer regions to be discriminated over large fields of view with high spatial resolution (Fig. 3a–c).

The structure-factor dependence of diffraction intensity, including its sensitivity to both layer number and stacking symmetry, has been quantitatively calculated and validated experimentally (Shevitski et al. 2013). These calculations demonstrated how specific diffraction conditions can enhance or suppress certain reflections depending on symmetry, and the experiments confirmed that these effects hold in real samples (Fig. 3d). Other reports also provided direct experimental evidence of layer-number–dependent contrast by showing that DF TEM images formed from the second-order Bragg reflection ($\:g=11\stackrel{-}{2}0$) (Fig. 3e, f) exhibit increased intensity with layer number up to approximately seven layers (Ping and Fuhrer 2012).

Although this approach has been most extensively developed and systematically applied to graphene, it is gradually being extended to other 2D materials. For example, three-dimensional electron diffraction patterns obtained under specimen tilt have enabled clear distinction between monolayer and multilayer in h-BN (Pan et al. 2012; Odlyzko and Mkhoyan, 2012) and α-RuCl_3_ (Yang et al. 2023). Similarly, the layer number of anisotropic black phosphorus has been roughly estimated from the intensity ratios among diffraction spots (Castellanos-Gomez et al. 2014). Together, these studies established both the theoretical framework and the experimental validation for using structure-factor selected reflections to map layer number and, under suitable conditions, stacking symmetry in 2D materials (Sung et al. 2019).

## Stacking order identification and domain boundary mapping

**Fig. 4:**
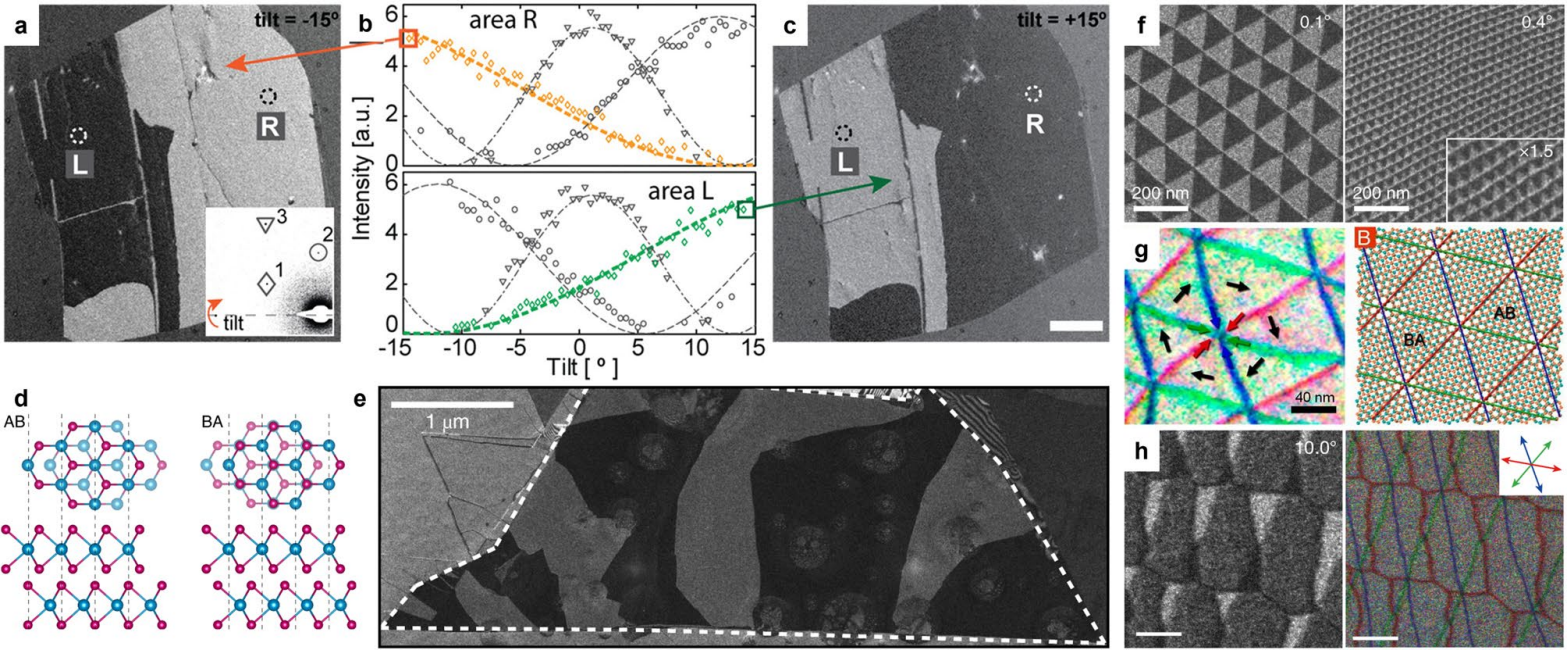
DF TEM analysis of stacking configurations and domain walls. (**a**) First-order DF image of bilayer graphene at − 15° tilt. (inset) SAED pattern of bilayer graphene. (**b**) Intensity variation with specimen tilt for two areas, R (upper panel) and L (lower panel). Each curve corresponds to the intensity of spots 1, 2, and 3 in the SAED pattern inset of (**a**), marked by diamond, circle, and triangle, respectively. (**c**) First-order DF image of bilayer graphene at + 15° tilt. Scale bar, 1 μm. Adapted with permission from (Brown et al., 2012). Copyright American Chemical Society. (**d**) Top and side views of AB (left) and BA (right) stacking orders in bilayer MoSe_2_. (**e**) First-order DF image of bilayer MoSe₂ with specimen tilt. Adapted with permission (Sung et al., 2020). Copyright Springer Nature. (**f**) First-order DF images of twisted bilayer graphene with twist angles of 0.1° (left) and 0.4° (right). Adapted with permission (Yoo et al., 2019). Copyright Springer Nature. (**g**) (Left) Domain wall map of twisted bilayer graphene constructed from second-order DF images, with red, blue, and green arrows indicating the directions of the Burgers vectors of domain walls. (Right) Corresponding atomic model. Adapted from Reference (Alden et al., 2013). (**h**) (Left) First-order DF image of twisted trilayer graphene and (right) domain wall map constructed from second-order DF images. Scale bar, 100 nm. Adapted with permission (Park et al., 2025). Copyright Springer Nature

In bilayer graphene, the most commonly studied stacking orders are Bernal (AB) and its mirror-symmetric counterpart (BA). Although these differ only by an in-plane translation and are electronically similar in flat samples, they can exhibit contrasting DF TEM intensities due to phase interference between electron waves diffracted from the two layers (Brown et al. 2012; Sung et al. 2019; Yoo et al. 2019; Alden et al. 2013; Lin et al. 2013; Butz et al. 2014). When a first-order Bragg reflection (e.g., $\:g=10\bar{1}0$) is used for imaging under exact zone-axis conditions, the path-length difference between scattered waves in AB and BA domains has the same magnitude but opposite sign, producing identical diffraction intensities and thus no DF contrast. Tilting the sample away from the zone axis modifies the phase term in the bilayer’s structure-factor such that one stacking moves toward more constructive interference while the other moves toward more destructive interference. At certain tilt angles, this difference is maximized, yielding strong stacking-dependent diffraction contrast (Fig. 4a-c). This principle, first demonstrated in DF TEM studies of bilayer graphene (Brown et al. 2012; Yoo et al. 2019; Alden et al. 2013; Lin et al. 2013; Butz et al. 2014), established that AB and BA domains can be unambiguously mapped over large areas with nanometer-scale resolution.

The same tilt-assisted DF TEM approach has been applied to other layered crystals with lower symmetries, including h-BN and TMDs such as MoS_2_ and WSe_2 _(Ko et al. 2023; Winkle et al. 2024; Kim et al. 2013; Rosenberger et al. 2020; Sung et al. 2020; Yang et al. 2024). In these systems, mirror-related domains, analogous to AB and BA in graphene and often labeled MX and XM (Fig. 4d), are observed. Here, M denotes the transition-metal atom (e.g., Mo or W), and X represents the chalcogen atom (e.g., S or Se). These domains can be distinguished using first-order Bragg reflections under optimized tilt conditions (Fig. 4e). This enables large-area mapping of stacking domains that influence valley polarization, interlayer exciton coupling, and ferroic ordering in TMDs multilayers.

In twisted 2D materials, DF TEM is a powerful tool for probing the reconstructed patterns that emerge from lattice relaxation. In these systems, periodic variations in local stacking order reorganize into low-energy domains whose distribution and connectivity critically influence the electronic and ferroic behavior. In small-angle twisted bilayer graphene, for example, relaxation produces triangular AB and BA domains separated by soliton-like boundaries that form an interconnected triangular network (Fig. 4f) (Yoo et al. 2019; Alden et al. 2013; Engelke et al. 2023). These boundaries can be directly visualized using second-order Bragg reflections such as those from the {$\:11\bar{2}0$} family, where their visibility depends on the relationship between the reciprocal lattice vector g and the Burgers vector b (or lattice-shift vector) describing the stacking change across the boundary (Yoo et al. 2019; Lin et al. 2013; Butz et al. 2014; Engelke et al. 2023). Imaging with multiple inequivalent second-order reflections allows for the identification of which boundary sets vanish in each image, enabling assignment of the Burgers vector to each family and quantitative mapping of the dislocation network (Fig. 4g).

In twisted trilayer graphene, multiple stacking sequences such as Bernal-type (ABA, ACA) and rhombohedral-type (ABC, ACB) can coexist with very small energy differences. This near-degeneracy drives complex reconstruction in which competing stackings form multi-scale domain structures. DF TEM is particularly effective for resolving these patterns, as shown by Park et al., (2025) where selective imaging of specific Bragg reflections revealed the spatial arrangement and connectivity of competing domains along with their boundary characteristics (Fig. 4h). Using first-order Bragg reflections under optimized tilt conditions, ABC, ACB, and Bernal stackings can be distinguished, while second-order reflections can be used to identify the Burgers vectors of the boundaries, thereby determining the specific stacking change across each domain wall. Such mapping is key to understanding how stacking configuration influences the electronic structure and the emergence of correlated ground states.

## Revealing lattice symmetry through Friedel pair breaking

**Fig. 5:**
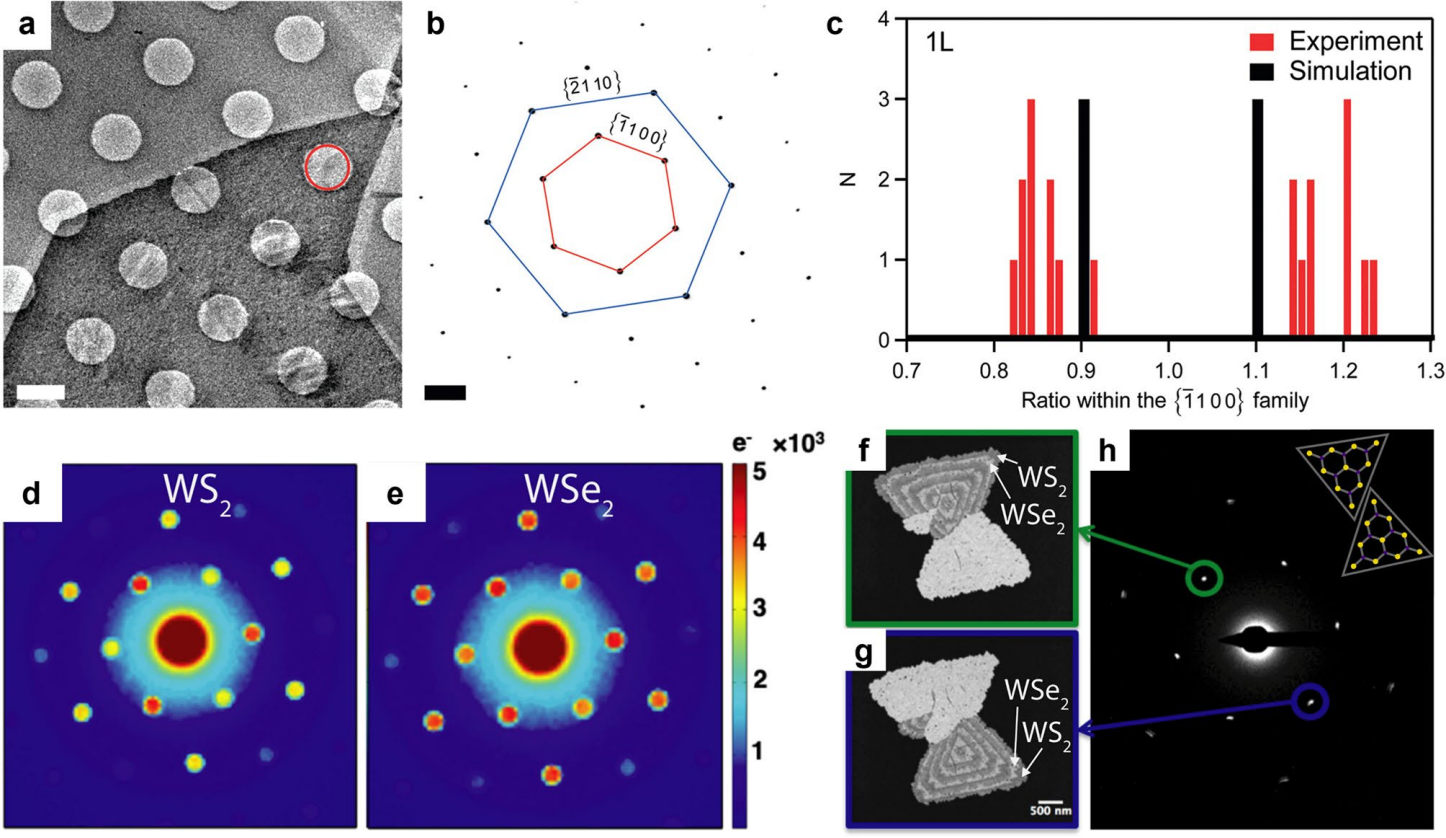
Polarity analysis through diffraction intensity. (**a**) Low-magnification TEM image of MoS₂. Scale bar, 1 μm. **(b)** SAED of the region marked with red circle in (a). Scale bar, 2 nm^− 1^. **(c)** Histogram of the intensity ratios between neighboring diffraction spots of the $\:\left\{\bar{1}100\right\}$ family in (b). Adapted with permission from (Brivio, et al., 2011). Copyright American Chemical Society. (**d-e**) Diffraction intensities of WS₂ (**d**) and WSe_2_ (**e**). (**f-h**) Polarity maps obtained by DF TEM, imaged from the diffraction spots marked with the green (**f**) and blue (**g**) circles in the SAED pattern (**h**), respectively, showing distinct contrasts. Adapted with permission (Deb et al., 2020). Copyright Elsevier

In monolayer TMDs with trigonal prismatic coordination, such as MoS_2_ (Fig. 5a) and WS_2_, the absence of an in-plane inversion center gives rise to a well-defined crystallographic polarity. This polarity, defined by the orientation of the metal–chalcogen sublattice, underpins valley-contrasting optical selection rules (Xiao et al. 2012; Mak et al. 2013) and layer-dependent ferroelectric phenomena (Wang et al. 2022a; Weston et al. 2022). In electron diffraction, polarity can be probed by comparing the intensities of a Friedel pair of reflections, g and − g. Under the assumptions of kinematic scattering and weak phase objects, Friedel’s law predicts these intensities to be identical, producing an inversion-symmetric diffraction pattern.

In practice, however, monolayer TMDs often exhibit Friedel pair breaking (Fig. 5b, c), where conjugate Bragg peaks display measurable intensity differences (van der Zande et al. 2013; Brivio et al. 2011; Deb et al. 2020; Ahn et al. 2024). Traditionally, such violations of Friedel’s law have been attributed to multiple scattering in thick, non-centrosymmetric crystals. Recent analysis and experiment have shown that even an atomically thin polar crystal can break Friedel symmetry when its in-plane inversion symmetry is absent (Deb et al. 2020). In this case, higher-order terms in the scattered wavefunction—representing multiple scattering paths that originate from the same atom—become significant, particularly for strong phase objects such as WS_2_ that contain heavy elements (Fig. 5d, e). These effects lead to polarity-dependent intensity asymmetry between g and − g, regardless of the incident beam energy.

By selecting an appropriate Bragg reflection sensitive to polarity and forming a DF TEM image from either g or − g, polar domains can be directly visualized over large areas. Regions of opposite polarity appear with inverted contrast when switching between the two conjugate reflections, enabling unambiguous domain mapping (Fig. 5f–h). This approach has been used to identify polar twins, rotational variants, and more complex polar textures in monolayer TMDs, providing a fast and non-destructive method for assessing polarity-related structural organization in 2D materials.

## In-situ/operando TEM

The combination of electron diffraction and DF TEM, when implemented under in-situ and operando conditions, offers a unique way to monitor the structural processes occurring in 2D materials under external stimuli. These techniques can distinguish structural variants such as different crystal phases (Zheng et al. 2022; Lee et al. 2023; Gavhane et al. 2021; Wang et al. 2022b), charge-density-wave (CDW) modulations (Danz et al. 2021; Sung et al. 2022b, 2024; Domröse et al. 2024; Durham et al. 2024), or stacking domains (Ko et al. 2023; Alden et al. 2013; Winkle et al. 2024; Zhang et al. 2024), all of which directly influence the functional properties of the material. Because such structural states can reorganize when exposed to temperature, electric fields, or other perturbations, capturing their evolution in real time is crucial for understanding and optimizing material performance. In-situ TEM allows the structural response to be tracked during controlled perturbations, while operando TEM extends this capability to devices under realistic working conditions, enabling direct correlation between structural evolution and functional output.

**Fig. 6:**
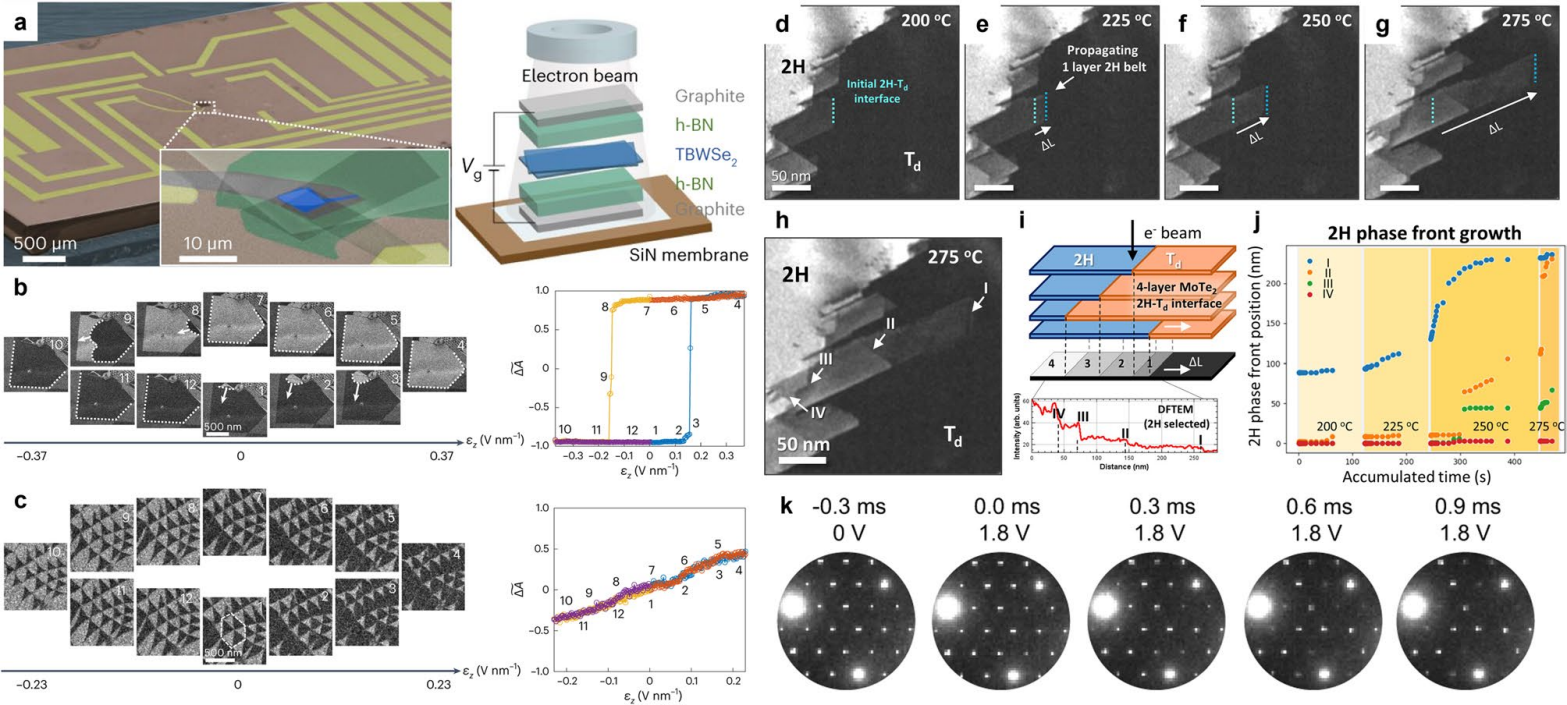
Domain evolution and phase transitions in 2D materials revealed by in-situ/operando TEM. (**a**) Scanning electron microscope (SEM) image (left) and schematic illustration (right) of the operando TEM device. (**b**) In-situ snapshots (left) of polar domains in bilayer WSe_2_ under electrical bias and the corresponding ferroelectric-like response (right). (**c**) Snapshots (left) of alternating polar domains in twisted bilayer WSe_2_ under bias, along with the corresponding antiferroelectric-like response (right). Adapted with permission (Ko et al., 2023). Copyright Springer Nature. (**d-g**) DF images of MoTe_2_ after applying a 0.5 s heat pulse at (**d**) 200 °C, (**e**) 225 °C, (**f**) 250 °C, and (**g**) 275 °C, showing the T_d_–2 H phase transition. (**h**) DF image of MoTe_2_ at 275 °C showing a layer-by-layer T_d_–2 H phase transition. Phase fronts of mono-, bi-, tri-, and quad-layers are labeled I–IV, respectively. (**i**) Schematic illustration of the T_d_–2 H interfaces and corresponding intensity profile illustrating the phase fronts. (**j**) Temporal evolution of 2 H phase fronts during pulsed heating. Adapted with permission from (Lee et al., 2023). Copyright American Chemical Society. (**k**) Evolution of the diffraction pattern in 1T-TaS_2_ during heating induced by a pulse applied at 0 ms. Adapted with permission (Hart et al., 2023). Copyright Springer Nature

In twisted h-BN and TMDs, unconventional forms of ferroelectric behavior have recently been reported, but the structural origins of these responses have remained unclear (Yasuda et al. 2021; Vizner Stern et al. 2021; Wang et al. 2022a; Weston et al. 2022). As described earlier, first-order DF TEM imaging can distinguish MX and XM stacking domains, which correspond to up- and down-polarized regions in these systems. While their static arrangement was well characterized, how these polar domains evolve under an applied electric field remained unknown, representing a key gap in linking structure to dielectric response. Ko et al. (2023) addressed this by fabricating a double-capacitor device geometry (Fig. 6a) and performing operando DF TEM imaging to follow, in real time, the reconfiguration of polar domains during electrical switching. Their measurements revealed that distinct modes of domain evolution correlate with either ferroelectric-like (Fig. 6b) or antiferroelectric-like responses (Fig. 6c), providing a clear structural origin for these behaviors and resolving prior ambiguities in the interpretation of interfacial sliding ferroelectricity. Because the temporal resolution of conventional DF TEM was insufficient to resolve the fastest domain wall motion, they used a stroboscopic measurement strategy to effectively bypass this limitation, enabling direct measurement of domain wall propagation speeds on short time scales and with the potential to extend the method into the sub-microsecond regime.

In another example, Lee et al. (2023) employed in-situ TEM to study the structural evolution of few-layer MoTe_2_ during its anisotropic layer-by-layer phase transition between the semiconducting 2 H phase and the semimetallic T_d_ phase (Fig. 6d-g). By combining diffraction and DF imaging, they were able to resolve the spatial progression of the transition front with high spatial and temporal resolution, revealing that the phase transformation proceeds through a sequence of discrete layer conversions rather than a uniform transformation (Fig. 6h-j). This insight clarified the microscopic pathway of a technologically important phase change relevant to phase-change memory and reconfigurable electronics.

Similarly, Hart et al. (2023) combined DF TEM with time-resolved electron diffraction to investigate how CDW order in 1T-TaS_2_ responds to applied electrical pulses (Fig. 6k). Their measurements captured the rapid suppression and reformation of the CDW superlattice, showing that defects play a decisive role in mediating the recovery pathway. This experiment demonstrated how structural modulations that govern correlated electronic states can be directly tracked under device-relevant driving, establishing a model for how to disentangle intrinsic order-parameter dynamics from extrinsic defect effects.

Beyond biasing and heating, in-situ DF TEM is increasingly integrated with other techniques. For example, in-situ cryogenic DF TEM was employed to visualize polar and phase domain walls in MoTe_2_, revealing conductive interfacial states between topologically distinct phases and demonstrating electron-beam–induced domain switching (Huang et al. 2019). In-situ straining DF TEM enabled modulation of phase and polarization in In_2_Se_3_, showing a reversible transition between ferroelectric and antiferroelectric phases under mechanical deformation (Zheng et al. 2022). DF ultrafast TEM combined with time-resolved SAED was utilized to capture photoinduced bulging and buckling dynamics in black phosphorus over nanosecond-to-microsecond timescales (Kim et al. 2020). These advances highlight the expanding role of in-situ TEM as a versatile platform for elucidating the structural dynamics of 2D materials and other quantum systems under external stimuli.

## Summary and outlook

Structural degrees of freedom in 2D van der Waals materials, including twist angle, stacking order, and out-of-plane corrugations, are key determinants of their electronic, optical, and ferroic properties. This review has highlighted how the combination of electron diffraction and DF TEM offers complementary reciprocal- and real-space perspectives for resolving these structural features and linking them to functionality.

Large-area DF TEM mapping is effective for identifying grain boundaries, rotational disorder, and stacking domain distributions, while tilt-assisted imaging can be applied more generally to distinguish different stacking orders in bilayer and multilayer systems. Diffraction-based analyses, such as tilt-series broadening measurements, can quantify out-of-plane distortions with high precision. Polarity mapping, achieved by detecting intensity asymmetries between Friedel-related reflections, enables direct visualization of polar domains in non-centrosymmetric crystals, which is essential for understanding polarity-dependent optical selection rules and interfacial ferroelectricity.

Extending these approaches to in-situ and operando experiments makes it possible to monitor structural dynamics during external stimuli, including electrical switching, thermally driven phase transitions, and CDW modulations. A current limitation of DF imaging is its temporal resolution, which is constrained by signal-to-noise considerations. This can be overcome by combining DF imaging with stroboscopic acquisition methods or integrating ultrafast TEM techniques, thereby enabling the capture of rapid domain wall motion and other fast structural processes.

Looking ahead, integrating four-dimensional STEM (4D-STEM) into in-situ and operando workflows will provide multi-channel structural information—such as strain, symmetry breaking, and phase coexistence—from a single dataset, greatly enhancing the efficiency of dynamic experiments. Coupling these high-dimensional datasets with machine learning will further accelerate analysis, enabling rapid, automated, and more sensitive detection of subtle structural changes. Together, these developments will expand the scope of diffraction and DF TEM from static structural mapping to real-time, multi-parameter tracking of structure–function relationships in 2D materials under realistic operating conditions.

## Data Availability

Not applicable.

## Abbreviations

2D: Two-dimensional
h-BN: Hexagonal boron nitride
TMDs: Transition metal dichalcogenides
TEM: Transmission electron microscopy
BF: Bright-field
DF: Dark-field
CVD: Chemical vapor deposition
SAED: Selected area electron diffraction
STEM: Scanning transmission electron microscopy
ADF-STEM: Annular dark-field scanning transmission electron microscopy
CDW: Charge-density-wave
SEM: Scanning electron microscopy
4D-STEM: Four-dimensional scanning transmission electron microscopy

## References

[CR1] G.H. Ahn et al., Strain-engineered growth of two-dimensional materials. Nat. Commun. **8**, 608 (2017) 28931806 10.1038/s41467-017-00516-5 PMC5606995

[CR2] S.J. Ahn et al., Dirac electrons in a dodecagonal graphene quasicrystal. Science **361**, 782–786 (2018) 29954987 10.1126/science.aar8412

[CR3] H. Ahn et al., Integrated 1D epitaxial mirror twin boundaries for ultrascaled 2D MoS₂ field-effect transistors. Nat. Nanotechnol. **19**, 955–961 (2024) 38961247 10.1038/s41565-024-01706-1

[CR4] J.S. Alden et al., Strain solitons and topological defects in bilayer graphene. Proc. Natl. Acad. Sci. U. S. A. **110**, 11256–11260 (2013) 23798395 10.1073/pnas.1309394110 PMC3710814

[CR5] A. Azizi et al., Dislocation motion and grain boundary migration in two-dimensional tungsten disulphide. Nat. Commun. **5**, 4867 (2014) 25202857 10.1038/ncomms5867

[CR6] R. Bistritzer, A.H. MacDonald, Moiré bands in twisted double-layer graphene. Proc. Natl. Acad. Sci. U. S. A. **108**, 12233–12237 (2011) 21730173 10.1073/pnas.1108174108 PMC3145708

[CR7] L. Britnell et al., Field-effect tunneling transistor based on vertical graphene heterostructures. Science **335**, 947–950 (2012a) 22300848 10.1126/science.1218461

[CR8] L. Britnell et al., Electron tunneling through ultrathin Boron nitride crystalline barriers. Nano Lett. **12**, 1707–1710 (2012b) 22380756 10.1021/nl3002205

[CR9] L. Britnell et al., Strong light-matter interactions in heterostructures of atomically thin films. Science **340**, 1311–1314 (2013) 23641062 10.1126/science.1235547

[CR10] J. Brivio, D.T.L. Alexander, A. Kis, Ripples and layers in ultrathin MoS₂ membranes. Nano Lett. **11**, 5148–5153 (2011) 22010987 10.1021/nl2022288

[CR11] M.S. Bronsgeest et al., Strain relaxation in CVD graphene: wrinkling with shear lag. Nano Lett. **15**, 5098–5104 (2015) 26171667 10.1021/acs.nanolett.5b01246

[CR12] L. Brown et al., Twinning and twisting of tri-and bilayer graphene. Nano Lett. **12**, 1609–1615 (2012) 22329410 10.1021/nl204547v

[CR13] B. Butz et al., Dislocations in bilayer graphene. Nature **505**, 533–537 (2014) 24352231 10.1038/nature12780

[CR14] Y. Cao et al., Correlated insulator behaviour at half-filling in magic-angle graphene superlattices. Nature **556**, 80–84 (2018a) 29512654 10.1038/nature26154

[CR15] Y. Cao et al., Unconventional superconductivity in magic-angle graphene superlattices. Nature **556**, 43–50 (2018b) 29512651 10.1038/nature26160

[CR16] A. Castellanos-Gomez et al., Isolation and characterization of few-layer black phosphorus. 2D Mater. **1**, 025001 (2014)

[CR17] E.V. Castro et al., Limits on charge carrier mobility in suspended graphene due to flexural phonons. Phys. Rev. Lett. **105**, 266601 (2010) 21231692 10.1103/PhysRevLett.105.266601

[CR18] F. Ceballos, M.Z. Bellus, H.-Y. Chiu, H. Zhao, Probing charge transfer excitons in a MoSe₂–WS₂ van der Waals heterostructure. Nanoscale **7**, 17523–17528 (2015) 26444979 10.1039/c5nr04723d

[CR19] H. Chen et al., Large-Area aligned growth of Low-Symmetry 2D ReS₂ on a High-Symmetry surface. ACS Nano. **18**, 35029–35038 (2024) 39658962 10.1021/acsnano.4c14162

[CR20] K.W. Clark et al., Spatially resolved mapping of electrical conductivity across individual domain (Grain) boundaries in graphene. ACS Nano **7**, 7956–7966 (2013) 23952068 10.1021/nn403056k

[CR21] H.J. Conley et al., Bandgap engineering of strained monolayer and bilayer MoS₂. Nano Lett. **13**, 3626–3630 (2013) 23819588 10.1021/nl4014748

[CR22] X. Cui et al., Multi-terminal transport measurements of MoS₂ using a Van der Waals heterostructure device platform. Nat. Nanotechnol. **10**, 534–540 (2015) 25915194 10.1038/nnano.2015.70

[CR23] T. Danz, T. Domröse, C. Ropers, Ultrafast nanoimaging of the order parameter in a structural phase transition. Science **371**, 371–374 (2021) 33479147 10.1126/science.abd2774

[CR24] C.R. Dean et al., Boron nitride substrates for high-quality graphene electronics. Nat. Nanotechnol. **5**, 722–726 (2010) 20729834 10.1038/nnano.2010.172

[CR25] C.R. Dean et al., Hofstadter’s butterfly and the fractal quantum hall effect in moiré superlattices. Nature **497**, 598–602 (2013) 23676673 10.1038/nature12186

[CR26] P. Deb et al., Imaging polarity in two dimensional materials by breaking friedel’s law. Ultramicroscopy **215**, 113019 (2020) 32521385 10.1016/j.ultramic.2020.113019

[CR27] Y. Deng et al., Gate-tunable room-temperature ferromagnetism in two-dimensional Fe₃GeTe₂. Nature. **563**, 94–99 (2018) 30349002 10.1038/s41586-018-0626-9

[CR28] T. Domröse et al., Nanoscale operando imaging of electrically driven charge-density wave phase transitions. Nano Lett. **24**, 12476–12485 (2024) 39316412 10.1021/acs.nanolett.4c03324 PMC11468880

[CR29] D. Dumcenco et al., Large-Area epitaxial monolayer MoS₂. ACS Nano. **9**, 4611–4620 (2015) 25843548 10.1021/acsnano.5b01281 PMC4415455

[CR30] D.B. Durham et al., Nanosecond structural dynamics during electrical melting of charge density waves in 1T-TaS₂. Phys. Rev. Lett. **132**, 226201 (2024) 38877909 10.1103/PhysRevLett.132.226201

[CR31] K. Elibol et al., Grain boundary-mediated nanopores in molybdenum disulfide grown by chemical vapor deposition. Nanoscale **9**, 1591–1598 (2017) 28070582 10.1039/c6nr08958e

[CR32] R. Engelke et al., Topological nature of dislocation networks in two-dimensional moiré materials. Phys. Rev. B **107**, 125413 (2023)

[CR33] X. Fan et al., Direct observation of grain boundaries in graphene through vapor hydrofluoric acid (VHF) exposure. Sci. Adv. **4**, eaar5170 (2018) 29806026 10.1126/sciadv.aar5170 PMC5969814

[CR34] H. Fang et al., Strong interlayer coupling in van der Waals heterostructures built from single-layer chalcogenides. Proc. Natl. Acad. Sci. U. S. A. **111**, 6198–6202 (2014) 24733906 10.1073/pnas.1405435111 PMC4035947

[CR35] A. Fasolino, J.H. Los, Katsnelson, M. I. Intrinsic ripples in graphene. Nat. Mater. **6**, 858–861 (2007) 17891144 10.1038/nmat2011

[CR36] L. Fei et al., Direct TEM observations of growth mechanisms of two-dimensional MoS₂ flakes. Nat. Commun. **7**, 12206 (2016) 27412892 10.1038/ncomms12206 PMC4947173

[CR37] Z. Fei et al., Ferroelectric switching of a two-dimensional metal. Nature **560**, 336–339 (2018) 30038286 10.1038/s41586-018-0336-3

[CR38] A.C. Ferrari et al., Raman spectrum of graphene and graphene layers. Phys. Rev. Lett. **97**, 187401 (2006) 17155573 10.1103/PhysRevLett.97.187401

[CR39] Y. Gao et al., Large-area synthesis of high-quality and uniform monolayer WS₂ on reusable Au foils. Nat. Commun. **6**, 8569 (2015a) 26450174 10.1038/ncomms9569 PMC4633959

[CR40] P. Gao, L. Wang, Y. Zhang, Y. Huang, K. Liu, Atomic-scale probing of the dynamics of sodium transport and intercalation-induced phase transformations in MoS₂. ACS Nano **9**, 11296–11301 (2015b) 26389724 10.1021/acsnano.5b04950

[CR41] D.S. Gavhane et al., In situ electron microscopy study of structural transformations in 2D CoSe₂. *npj 2D Materials and Applications* 5, 24 (2021)

[CR42] A.K. Geim, I.V. Grigorieva, Van der Waals heterostructures. Nature **499**, 419–425 (2013) 23887427 10.1038/nature12385

[CR43] D. Geng et al., Uniform hexagonal graphene flakes and films grown on liquid copper surface. Proc. Natl. Acad. Sci. U. S. A. **109**, 7992–7996 (2012) 22509001 10.1073/pnas.1200339109 PMC3361379

[CR44] Y. Gong et al., Vertical and in-plane heterostructures from WS₂/MoS₂ monolayers. Nat. Mater. **13**, 1135–1142 (2014) 25262094 10.1038/nmat4091

[CR45] C. Gong et al., Discovery of intrinsic ferromagnetism in two-dimensional van der Waals crystals. Nature **546**, 265–269 (2017) 28445468 10.1038/nature22060

[CR46] C. Gong, E.M. Kim, Y. Wang, G. Lee, X. Zhang, Multiferroicity in atomic van der Waals heterostructures. Nat. Commun. **10**, 2657 (2019) 31201316 10.1038/s41467-019-10693-0 PMC6570651

[CR47] S.J. Haigh et al., Cross-sectional imaging of individual layers and buried interfaces of graphene-based heterostructures and superlattices. Nat. Mater. **11**, 764–767 (2012) 22842512 10.1038/nmat3386

[CR48] Y. Han et al., Strain mapping of two-dimensional heterostructures with subpicometer precision. Nano Lett. **18**, 3746–3751 (2018a) 29775315 10.1021/acs.nanolett.8b00952

[CR49] Y. Han et al., Sub-nanometre channels embedded in two-dimensional materials. Nat. Mater. **17**, 129–133 (2018b) 29200195 10.1038/nmat5038

[CR50] J.L. Hart et al., In Operando cryo-STEM of pulse-induced charge density wave switching in TaS₂. Nat. Commun. **14**, 8202 (2023) 38081844 10.1038/s41467-023-44093-2 PMC10713631

[CR51] X. He et al., Strain engineering in monolayer WS₂, MoS₂, and the WS₂/MoS₂ heterostructure. Appl. Phys. Lett. **109**, 173105 (2016)

[CR52] O. Hod, Interlayer commensurability and superlubricity in rigid layered materials. Phys. Rev. B **86**, 075444 (2012)

[CR53] X. Hong et al., Ultrafast charge transfer in atomically thin MoS₂/WS₂ heterostructures. Nat. Nanotechnol. **9**, 682–686 (2014) 25150718 10.1038/nnano.2014.167

[CR54] P.Y. Huang et al., Grains and grain boundaries in single-layer graphene atomic patchwork quilts. Nature **469**, 389–392 (2011) 21209615 10.1038/nature09718

[CR55] B. Huang et al., Layer-dependent ferromagnetism in a Van der Waals crystal down to the monolayer limit. Nature **546**, 270–273 (2017) 28593970 10.1038/nature22391

[CR56] F.-T. Huang et al., Polar and phase domain walls with conducting interfacial States in a Weyl semimetal MoTe₂. Nat. Commun. **10**, 4211 (2019) 31527602 10.1038/s41467-019-11949-5 PMC6746811

[CR57] B. Hunt et al., Massive Dirac fermions and Hofstadter butterfly in a Van der Waals heterostructure. Science **340**, 1427–1430 (2013) 23686343 10.1126/science.1237240

[CR58] M. Ishigami, J.H. Chen, W.G. Cullen, M.S. Fuhrer, E.D. Williams, Atomic structure of graphene on SiO₂. Nano Lett. **7**, 1643–1648 (2007) 17497819 10.1021/nl070613a

[CR59] J.-W. Jiang, H.S. Park, Mechanical properties of MoS₂/graphene heterostructures. Appl. Phys. Lett. **105**, 033108 (2014)

[CR60] J. Jiang et al., Chirality-transferred epitaxy of circular polarization-sensitive ReS₂ monolayer single crystals. Nat. Commun. **16**, 7119 (2025) 40753076 10.1038/s41467-025-61849-0 PMC12318066

[CR61] W. Jin et al., Direct measurement of the thickness-dependent electronic band structure of MoS₂ using angle-resolved photoemission spectroscopy. Phys. Rev. Lett. **111**, 106801 (2013) 25166690 10.1103/PhysRevLett.111.106801

[CR62] C. Jin et al., Observation of moiré excitons in WSe₂/WS₂ heterostructure superlattices. Nature. **567**, 76–80 (2019) 30804525 10.1038/s41586-019-0976-y

[CR63] K. Kim et al., Grain boundary mapping in polycrystalline graphene. ACS Nano **5**, 2142–2146 (2011) 21280616 10.1021/nn1033423

[CR64] C.-J. Kim et al., Stacking order dependent second harmonic generation and topological defects in h-BN bilayers. Nano Lett. **13**, 5660–5665 (2013) 24125021 10.1021/nl403328s

[CR65] K. Kim et al., Selective metal deposition at graphene line defects by atomic layer deposition. Nat. Commun. **5**, 4781 (2014) 25179368 10.1038/ncomms5781

[CR66] K. Kim et al., Van der Waals heterostructures with high accuracy rotational alignment. Nano Lett. **16**, 1989–1995 (2016) 26859527 10.1021/acs.nanolett.5b05263

[CR67] H. Kim et al., Synthetic WSe₂ monolayers with high photoluminescence quantum yield. Sci. Adv. **5**, eaau4728 (2019) 30613771 10.1126/sciadv.aau4728 PMC6314873

[CR68] Y.-J. Kim, Y. Lee, K. Kim, O.-H. Kwon, Light-induced anisotropic morphological dynamics of black phosphorus membranes visualized by dark-field ultrafast electron microscopy. ACS Nano **14**, 11383–11393 (2020) 32790334 10.1021/acsnano.0c03644

[CR69] D.A. Kirilenko, A.T. Dideykin, G. Van Tendeloo, Measuring the corrugation amplitude of suspended and supported graphene. Phys. Rev. B **84**, 235417 (2011)

[CR70] K. Ko et al., Operando electron microscopy investigation of polar domain dynamics in twisted Van der Waals homobilayers. Nat. Mater. **22**, 992–998 (2023) 37365226 10.1038/s41563-023-01595-0

[CR71] A. Laitinen et al., Electron–phonon coupling in suspended graphene: supercollisions by ripples. Nano Lett. **14**, 3009–3013 (2014) 24842236 10.1021/nl404258a

[CR72] T. Latychevskaia, C. Escher, H.-W. Fink, Moiré structures in twisted bilayer graphene studied by transmission electron microscopy. Ultramicroscopy **197**, 46–52 (2019) 30496888 10.1016/j.ultramic.2018.11.009

[CR73] C. Lee, X. Wei, J.W. Kysar, J. Hone, Measurement of the elastic properties and intrinsic strength of monolayer graphene. Science **321**, 385–388 (2008) 18635798 10.1126/science.1157996

[CR74] G.-H. Lee et al., High-strength chemical-vapor–deposited graphene and grain boundaries. Science **340**, 1073–1076 (2013) 23723231 10.1126/science.1235126

[CR75] C.-H. Lee et al., In situ imaging of an anisotropic Layer-by-Layer phase transition in Few-Layer MoTe₂. Nano Lett. **23**, 677–684 (2023) 36648125 10.1021/acs.nanolett.2c04550

[CR76] L. Li, M. Wu, Binary compound bilayer and multilayer with vertical polarizations: Two-Dimensional Ferroelectrics, Multiferroics, and nanogenerators. ACS Nano. **11**, 6382–6388 (2017) 28602074 10.1021/acsnano.7b02756

[CR77] G. Li et al., Observation of van Hove singularities in twisted graphene layers. Nat. Phys. **6**, 109–113 (2010)

[CR78] Y. Li et al., Probing symmetry properties of Few-Layer MoS₂ and h-BN by optical Second-Harmonic generation. Nano Lett. **13**, 3329–3333 (2013) 23718906 10.1021/nl401561r

[CR79] L. Li et al., Black phosphorus field-effect transistors. Nat. Nanotechnol. **9**, 372–377 (2014a) 24584274 10.1038/nnano.2014.35

[CR80] Y. Li et al., Measurement of the optical dielectric function of monolayer transition-metal dichalcogenides: MoS₂, MoSe₂, WS₂, and WSe₂. Phys. Rev. B **90**, 205422 (2014b)

[CR81] J. Lin et al., AC/AB stacking boundaries in bilayer graphene. Nano Lett. **13**, 3262–3268 (2013) 23772750 10.1021/nl4013979

[CR82] K. Liu et al., Evolution of interlayer coupling in twisted molybdenum disulfide bilayers. Nat. Commun. **5**, 4966 (2014a) 25233054 10.1038/ncomms5966

[CR83] K. Liu et al., Elastic properties of Chemical-Vapor-Deposited monolayer MoS₂, WS₂, and their bilayer heterostructures. Nano Lett. **14**, 5097–5103 (2014b) 25120033 10.1021/nl501793a

[CR84] X. Liu et al., Tunable spin-polarized correlated states in twisted double bilayer graphene. Nature **583**, 221–225 (2020) 32641816 10.1038/s41586-020-2458-7

[CR85] A. Luican et al., Single-layer behavior and its breakdown in twisted graphene layers. Phys. Rev. Lett. **106**, 126802 (2011) 21517338 10.1103/PhysRevLett.106.126802

[CR86] T.H. Ly et al., Misorientation-angle-dependent electrical transport across molybdenum disulfide grain boundaries. Nat. Commun. **7**, 10426 (2016) 26813605 10.1038/ncomms10426 PMC4737806

[CR87] K.F. Mak, C. Lee, J. Hone, J. Shan, T.F. Heinz, Atomically thin MoS₂: a new direct-gap semiconductor. Phys. Rev. Lett. **105**, 136805 (2010) 21230799 10.1103/PhysRevLett.105.136805

[CR88] K.F. Mak et al., Tightly bound Trions in monolayer MoS₂. Nat. Mater. **12**, 207–211 (2013) 23202371 10.1038/nmat3505

[CR89] E. Mariani, von F. Oppen, Flexural phonons in Free-Standing graphene. Phys. Rev. Lett. **100**, 076801 (2008) 18352583 10.1103/PhysRevLett.100.076801

[CR90] P. Meng et al., Sliding induced multiple polarization states in two-dimensional ferroelectrics. Nat. Commun. **13**, 7696 (2022) 36509811 10.1038/s41467-022-35339-6 PMC9744910

[CR91] J.C. Meyer et al., On the roughness of single-and bi-layer graphene membranes. Solid State Commun. **143**, 101–109 (2007a)

[CR92] J.C. Meyer et al., The structure of suspended graphene sheets. Nature **446**, 60–63 (2007b) 17330039 10.1038/nature05545

[CR93] T.M.G. Mohiuddin et al., Uniaxial strain in graphene by Raman spectroscopy: G peak splitting, Grüneisen parameters, and sample orientation. Phys. Rev. B **79**, 205433 (2009)

[CR94] R.R. Nair et al., Fine structure constant defines visual transparency of graphene. Science **320**, 1308–1308 (2008) 18388259 10.1126/science.1156965

[CR95] H.K. Ng et al., Improving carrier mobility in two-dimensional semiconductors with rippled materials. Nat. Electron. **5**, 489–496 (2022)

[CR96] K.S. Novoselov et al., Electric field effect in atomically thin carbon films. Science. **306**, 666–669 (2004) 15499015 10.1126/science.1102896

[CR97] A.N. Obraztsov, E.A. Obraztsova, A.V. Tyurnina, A.A. Zolotukhin, Chemical vapor deposition of thin graphite films of nanometer thickness. Carbon **45**, 2017–2021 (2007)

[CR98] M.L. Odlyzko, K.A. Mkhoyan, Identifying hexagonal boron nitride monolayers by transmission electron microscopy. Microsc. Microanal. **18**, 558–567 (2012) 22640966 10.1017/S143192761200013X

[CR99] C.T. Pan et al., Nanoscale electron diffraction and plasmon spectroscopy of single- and few-layer Boron nitride. Phys. Rev. B **85**, 045440 (2012)

[CR100] J.M. Park, Y. Cao, K. Watanabe, T. Taniguchi, P. Jarillo-Herrero, Tunable strongly coupled superconductivity in magic-angle twisted trilayer graphene. Nature **590**, 249–255 (2021) 33526935 10.1038/s41586-021-03192-0

[CR101] D. Park et al., Unconventional domain tessellations in moiré-of-moiré lattices. Nature **641**, 896–903 (2025) 40369071 10.1038/s41586-025-08932-0

[CR102] V.M. Pereira, A.H. Castro Neto, Strain engineering of graphene’s electronic structure. Phys. Rev. Lett. **103**, 046801 (2009) 19659379 10.1103/PhysRevLett.103.046801

[CR103] J. Ping, M.S. Fuhrer, Layer number and stacking sequence imaging of few-layer graphene by transmission electron microscopy. Nano Lett. **12**, 4635–4641 (2012) 22873797 10.1021/nl301932v

[CR104] L.A. Ponomarenko et al., Cloning of Dirac fermions in graphene superlattices. Nature **497**, 594–597 (2013) 23676678 10.1038/nature12187

[CR105] H.I. Rasool et al., Atomic-scale characterization of graphene grown on copper (100) single crystals. J. Am. Chem. Soc. **133**, 12536–12543 (2011) 21732685 10.1021/ja200245p

[CR106] E.C. Regan et al., Mott and generalized wigner crystal States in WSe₂/WS₂ moiré superlattices. Nature. **579**, 359–363 (2020) 32188951 10.1038/s41586-020-2092-4

[CR107] P. Rivera et al., Observation of long-lived interlayer excitons in monolayer MoSe₂–WSe₂ heterostructures. Nat. Commun. **6**, 6242 (2015) 25708612 10.1038/ncomms7242

[CR108] P. Rivera et al., Valley-polarized exciton dynamics in a 2D semiconductor heterostructure. Science **351**, 688–691 (2016) 26912854 10.1126/science.aac7820

[CR109] Y. Rong et al., Controlled preferential oxidation of grain boundaries in monolayer tungsten disulfide for direct optical imaging. ACS Nano **9**, 3695–3703 (2015) 25870912 10.1021/acsnano.5b00852

[CR110] M.R. Rosenberger et al., Twist Angle-Dependent atomic reconstruction and Moiré patterns in transition metal dichalcogenide heterostructures. ACS Nano. **14**, 4550–4558 (2020) 32167748 10.1021/acsnano.0c00088

[CR111] Y. Saito, J. Ge, K. Watanabe, T. Taniguchi, A.F. Young, Independent superconductors and correlated insulators in twisted bilayer graphene. Nat. Phys. **16**, 926–930 (2020)

[CR112] F. Shao et al., Substrate influence on transition metal dichalcogenide monolayer exciton absorption linewidth broadening. Phys. Rev. Mater. **6**, 074005 (2022)

[CR113] A.L. Sharpe et al., Emergent ferromagnetism near three-quarters filling in twisted bilayer graphene. Science **365**, 605–608 (2019) 31346139 10.1126/science.aaw3780

[CR114] B. Shevitski et al., Dark-field transmission electron microscopy and the Debye-Waller factor of graphene. Phys. Rev. B **87**, 045417 (2013) 10.1103/PhysRevB.87.045417 PMC4167771 25242882

[CR115] L. Song et al., Large scale growth and characterization of atomic hexagonal boron nitride layers. Nano Lett. **10**, 3209–3215 (2010) 20698639 10.1021/nl1022139

[CR116] H.S. Song et al., Origin of the relatively low transport mobility of graphene grown through chemical vapor deposition. Sci. Rep. **2**, 337 (2012) 22468224 10.1038/srep00337 PMC3313616

[CR117] A. Splendiani et al., Emerging photoluminescence in monolayer MoS₂. Nano Lett. **10**, 1271–1275 (2010) 20229981 10.1021/nl903868w

[CR118] F. Sui et al., Sliding ferroelectricity in van der Waals layered γ-InSe semiconductor. Nat. Commun. **14**, 36 (2023) 36596789 10.1038/s41467-022-35490-0 PMC9810696

[CR119] S.H. Sung, N. Schnitzer, L. Brown, J. Park, R. Hovden, Stacking, strain, and twist in 2D materials quantified by 3D electron diffraction. Phys. Rev. Mater. **3**, 064003 (2019)

[CR120] J. Sung et al., Broken mirror symmetry in excitonic response of reconstructed domains in twisted MoSe₂/MoSe₂ bilayers. Nat. Nanotechnol. **15**, 750–754 (2020) 32661373 10.1038/s41565-020-0728-z

[CR121] S.H. Sung et al., Torsional periodic lattice distortions and diffraction of twisted 2D materials. Nat. Commun. **13**, 7826 (2022a) 36535920 10.1038/s41467-022-35477-x PMC9763474

[CR122] S.H. Sung et al., Two-dimensional charge order stabilized in clean polytype heterostructures. Nat. Commun. **13**, 413 (2022b) 35058434 10.1038/s41467-021-27947-5 PMC8776735

[CR123] S.H. Sung et al., Endotaxial stabilization of 2D charge density waves with long-range order. Nat. Commun. **15**, 1403 (2024) 38360698 10.1038/s41467-024-45711-3 PMC10869719

[CR124] S.S. Sunku et al., Photonic crystals for nano-light in moiré graphene superlattices. Science **362**, 1153–1156 (2018) 30523109 10.1126/science.aau5144

[CR125] K. Thodkar, M. Plodinec, F. Gramm, K. Kunze, Probing the intrinsic strain in suspended graphene films using electron and optical microscopy. Adv. Sci. **11**, 2305366 (2024) 10.1002/advs.202305366 PMC10837373 38054210

[CR126] J.D. Thomsen et al., Suppression of intrinsic roughness in encapsulated graphene. Phys. Rev. B **96**, 014101 (2017)

[CR127] S. Tongay et al., Tuning interlayer coupling in Large-Area heterostructures with CVD-Grown MoS₂ and WS₂ monolayers. Nano Lett. **14**, 3185–3190 (2014) 24845201 10.1021/nl500515q

[CR128] A.W. Tsen et al., Tailoring electrical transport across grain boundaries in polycrystalline graphene. Science **336**, 1143–1146 (2012) 22654054 10.1126/science.1218948

[CR129] A.W. Tsen et al., Structure and control of charge density waves in two-dimensional 1T-TaS₂. *Proceedings of the National Academy of Sciences* 112, 15054–15059 (2015) 10.1073/pnas.1512092112 PMC4679066 26598707

[CR130] van der A.M. Zande et al., Grains and grain boundaries in highly crystalline monolayer molybdenum disulphide. Nat. Mater. **12**, 554–561 (2013) 23644523 10.1038/nmat3633

[CR131] Van M. Winkle et al., Engineering interfacial polarization switching in Van der Waals multilayers. Nat. Nanotechnol. **19**, 751–757 (2024) 38504024 10.1038/s41565-024-01642-0

[CR132] M. Vizner Stern et al., Interfacial ferroelectricity by van der Waals sliding. Science **372**, 1462–1466 (2021) 10.1126/science.abe8177 34112727

[CR133] L. Wang et al., Correlated electronic phases in twisted bilayer transition metal dichalcogenides. Nat. Mater. **19**, 861–866 (2020) 32572205 10.1038/s41563-020-0708-6

[CR134] X. Wang et al., Interfacial ferroelectricity in rhombohedral-stacked bilayer transition metal dichalcogenides. Nat. Nanotechnol. **17**, 367–371 (2022a) 35039684 10.1038/s41565-021-01059-z

[CR135] Y. Wang et al., Atomistic observation of the local phase transition in MoTe₂ for application in homojunction photodetectors. Small **18**, 2200913 (2022b) 10.1002/smll.202200913 35411673

[CR136] A. Weston et al., Interfacial ferroelectricity in marginally twisted 2D semiconductors. Nat. Nanotechnol. **17**, 390–395 (2022) 35210566 10.1038/s41565-022-01072-w PMC9018412

[CR137] C.R. Woods et al., Commensurate–incommensurate transition in graphene on hexagonal Boron nitride. Nat. Phys. **10**, 451–456 (2014)

[CR138] Y. Wu et al., A Van der Waals interface hosting two groups of magnetic skyrmions. Adv. Mater. **34**, 2110583 (2022) 10.1002/adma.202110583 35218078

[CR139] X. Xi et al., Ising pairing in superconducting NbSe₂ atomic layers. Nat. Phys. **12**, 139–143 (2016)

[CR140] D. Xiao, G.-B. Liu, W. Feng, X. Xu, W. Yao, Coupled spin and valley physics in monolayers of MoS₂ and other Group-VI dichalcogenides. Phys. Rev. Lett. **108**, 196802 (2012) 23003071 10.1103/PhysRevLett.108.196802

[CR141] S. Xie et al., Coherent, atomically thin transition-metal dichalcogenide superlattices with engineered strain. Science **359**, 1131–1136 (2018) 29590041 10.1126/science.aao5360

[CR142] P. Xu et al., Unusual ultra-low-frequency fluctuations in freestanding graphene. Nat. Commun. **5**, 3720 (2014) 24770734 10.1038/ncomms4720

[CR143] B. Yang et al., Magnetic anisotropy reversal driven by structural symmetry-breaking in monolayer α-RuCl₃. Nat. Mater. **22**, 50–57 (2023) 36396963 10.1038/s41563-022-01401-3

[CR144] T.H. Yang et al., Ferroelectric transistors based on shear-transformation-mediated rhombohedral-stacked molybdenum disulfide. Nat. Electron. **7**, 29–38 (2024)

[CR145] M. Yankowitz et al., Emergence of superlattice Dirac points in graphene on hexagonal Boron nitride. Nat. Phys. **8**, 382–386 (2012)

[CR146] M. Yankowitz et al., Tuning superconductivity in twisted bilayer graphene. Science **363**, 1059–1064 (2019) 30679385 10.1126/science.aav1910

[CR147] P. Yasaei et al., Chemical sensing with switchable transport channels in graphene grain boundaries. Nat. Commun. **5**, 4911 (2014) 25241799 10.1038/ncomms5911

[CR148] K. Yasuda, X. Wang, K. Watanabe, T. Taniguchi, P. Jarillo-Herrero, Stacking-engineered ferroelectricity in bilayer Boron nitride. Science **372**, 1458–1462 (2021) 10.1126/science.abd3230 34045323

[CR149] O.V. Yazyev, S.G. Louie, Topological defects in graphene: dislocations and grain boundaries. Phys. Rev. B **81**, 195420 (2010)

[CR150] H. Yoo et al., Atomic and electronic reconstruction at the Van der Waals interface in twisted bilayer graphene. Nat. Mater. **18**, 448–453 (2019) 30988451 10.1038/s41563-019-0346-z

[CR151] M. Yoshida, R. Suzuki, Y. Zhang, M. Nakano, Y. Iwasa, Memristive phase switching in two-dimensional 1T-TaS₂ crystals. Sci. Adv. **1**, e1500606 (2015) 26601295 10.1126/sciadv.1500606 PMC4646809

[CR152] Q. Yu et al., Control and characterization of individual grains and grain boundaries in graphene grown by chemical vapour deposition. Nat. Mater. **10**, 443–449 (2011) 21552269 10.1038/nmat3010

[CR153] J.M. Yuk et al., Superstructural defects and superlattice domains in stacked graphene. Carbon **80**, 755–761 (2014)

[CR154] Y. Zhang, J.P. Small, W.V. Pontius, P. Kim, Fabrication and electric-field-dependent transport measurements of mesoscopic graphite devices. Appl. Phys. Lett. **86**, 073104 (2005)

[CR155] Y. Zhang et al., Direct observation of a widely tunable bandgap in bilayer graphene. Nature **459**, 820–823 (2009) 19516337 10.1038/nature08105

[CR156] S. Zhang et al., Extraordinary photoluminescence and strong temperature/angle-dependent Raman responses in few-layer phosphorene. ACS Nano **8**, 9590–9596 (2014) 25188827 10.1021/nn503893j

[CR157] Y. Zhang et al., Atom-by-atom imaging of moiré transformations in 2D transition metal dichalcogenides. Sci. Adv. **10**, eadk1874 (2024) 38536909 10.1126/sciadv.adk1874 PMC10971488

[CR158] W. Zhao et al., Evolution of electronic structure in atomically thin sheets of WS₂ and WSe₂. ACS Nano. **7**, 791–797 (2013) 23256505 10.1021/nn305275h

[CR159] Z. Zheng et al., Unconventional ferroelectricity in moiré heterostructures. Nature **588**, 71–76 (2020) 33230334 10.1038/s41586-020-2970-9

[CR160] X. Zheng et al., Phase and polarization modulation in two-dimensional In₂Se₃ via in situ transmission electron microscopy. Sci. Adv. **8**, eabo0773 (2022) 36269828 10.1126/sciadv.abo0773 PMC9586485

[CR161] D. Zhong et al., Van der Waals engineering of ferromagnetic semiconductor heterostructures for spin and valleytronics. Sci. Adv. **3**, e1603113 (2017) 28580423 10.1126/sciadv.1603113 PMC5451195

[CR162] H. Zhou et al., Large area growth and electrical properties of p-Type WSe₂ atomic layers. Nano Lett. **15**, 709–713 (2015) 25434747 10.1021/nl504256y PMC4296926

[CR163] C. Zhou et al., Carrier type control of WSe₂ Field-Effect transistors by thickness modulation and MoO₃ layer doping. Adv. Funct. Mater. **26**, 4223–4230 (2016)

[CR164] J. Zhu et al., Boundary activated hydrogen evolution reaction on monolayer MoS₂. Nat. Commun. **10**, 1348 (2019) 30902982 10.1038/s41467-019-09269-9 PMC6430794

